# Clues to unraveling the coral species problem: distinguishing species from geographic variation in *Porites* across the Pacific with molecular markers and microskeletal traits

**DOI:** 10.7717/peerj.751

**Published:** 2015-02-03

**Authors:** Zac Forsman, Gerrard M. Wellington, George E. Fox, Robert J. Toonen

**Affiliations:** 1 Hawai‘i Institute of Marine Biology, Kāne‘ohe, HI, USA; 2 Department of Biology and Biochemistry, University of Houston, Houston, TX, USA

**Keywords:** Porites, ITS region, Species delimitation, Coral reef, Micro-morphology, Identification

## Abstract

Morphological variation in the geographically widespread coral *Porites lobata* can make it difficult to distinguish from other massive congeneric species. This morphological variation could be attributed to geographic variability, phenotypic plasticity, or a combination of such factors. We examined genetic and microscopic morphological variability in *P. lobata* samples from the Galápagos, Easter Island, Tahiti, Fiji, Rarotonga, and Australia. Panamanian *P. evermanni* specimens were used as a previously established distinct outgroup against which to test genetic and morphological methods of discrimination. We employed a molecular analysis of variance (AMOVA) based on ribosomal internal transcribed spacer region (ITS) sequence, principal component analysis (PCA) of skeletal landmarks, and Mantel tests to compare genetic and morphological variation. Both genetic and morphometric methods clearly distinguished *P. lobata* and *P. evermanni*, while significant genetic and morphological variance was attributed to differences among geographic regions for *P. lobata*. Mantel tests indicate a correlation between genetic and morphological variation for *P. lobata* across the Pacific. Here we highlight landmark morphometric measures that correlate well with genetic differences, showing promise for resolving species of *Porites*, one of the most ubiquitous yet challenging to identify architects of coral reefs.

## Introduction

Corals form the foundation of an ecosystem that is iconic for complexity, biodiversity, and dramatic global decline (e.g., Hughes et al., 2003; Carpenter et al., 2008; De’ath, Lough & Fabricius, 2009). More than a half billion people across the globe rely directly on coral reef ecosystems as a significant source of their diet, many more recognize the great intrinsic biological and cultural value of these ecosystems, and the economic benefits of coral reefs to local and global economies are well-documented (Wilkinson, 2008). It is increasingly important to understand biodiversity before it is permanently lost; however, evaluating extinction risk for reef building corals is extremely problematic, due to taxonomic uncertainty and a lack of understanding of species boundaries (Brainard et al., 2011). Coral species boundaries are poorly understood and hybridization, recent speciation, phenotypic polymorphism, and phenotypic plasticity may all contribute to taxonomic confusion. Corals have baffled taxonomists for centuries, and recent genetic work has uncovered striking examples of convergent or parallel evolution (Fukami et al., 2004; Forsman et al., 2009), extreme phenotypic variability and plasticity (Todd, 2008; Marti-Puig et al., 2014), sibling or cryptic species (Forsman & Birkeland, 2009; Forsman et al., 2010; Stefani et al., 2011), and overlap between intraspecific and interspecific morphological variation (Forsman et al., 2006; Forsman et al., 2009; Stat et al., 2012). The combination of molecular genetics and morphological characters provides a promising path toward ending much of the confusion in scleractinian systematics (Budd et al., 2010).

The genus *Porites* (Link, 1807) has long been a prime example of ‘the species problem’ due to complex patterns of morphological variation (Vaughan, 1907; Brakel, 1977). The genus has been one of the most important and abundant reef-building corals over the last 20 million years (Frost, 1977), leaving behind an excellent yet difficult to interpret fossil record (Zlatarski, 2010). Species of *Porites* have among the highest dispersal potentials (Fadlallah, 1983; Harrison, 2011) and largest geographic ranges, and the genus is one of very few to occur worldwide in the tropics (Veron & Stafford-Smith, 2000). Mounding *Porites* species are a preferred model organism for paleoclimate studies e.g., Wellington & Dunbar (1995) and Rosenfeld et al. (2003), due to annual growth bands that preserve seawater isotopes in massive colonies approaching hundreds or even a thousand years of age (Brown et al., 2009). Despite the fact that *Porites* is relatively well studied, species boundaries remain poorly understood and are the subject of ongoing debate (Brakel, 1977; Jameson, 1997; Forsman et al., 2009; Jameson & Cairns, 2012; Prada et al., 2014).

Scleractinian taxonomy is based on morphological and skeletal architecture, and the genus *Porites* is renowned as particularly challenging to identify both in the field and in the laboratory *Porites* corallites are small, irregular, and highly variable, and colony level morphology can range from massive to branching within several well-resolved genetic clades (Forsman et al., 2009). Transplantation studies have shown that at least one species (*P. sillimaniani*) can grow in plates or branches depending on depth (Muko et al., 2000). High variability in colony and corallite level skeletal characteristics is typified by the most widely distributed species *P. lobata* (Dana, 1846). *P. lobata* occurs in a wide variety of habitats over an enormous geographic range, spanning much of the entire Pacific and Indian Oceans. Colony and corallite level characteristics vary geographically, which has led to numerous named ‘formae,’ ‘subformae’ and synonyms (Bernard, 1902; Vaughan, 1907; Hoffmeister, 1926; Veron, Pichon & Wijsman-Best, 1977; Veron & Stafford-Smith, 2000). Colony morphology ranges from encrusting, plate-like or bolder-like forms, to thin protruding lobe, fin or columner forms. *P. lobata* is also a member of a large genetic species complex that includes branching morphospecies such as *P. compressa*, *P. cylindrica*, *P. annae*, and *P. duerdeni*, (Forsman et al., 2009). Interestingly, these branching varieties are not found on Eastern Pacific reefs but are prevalent in the Central and Western Pacific. Factors contributing to these patterns of morphological variation may include: phenotypic plasticity in response to environmental or ecological conditions; geographic isolation and genetic drift; hybridization between previously isolated lineages; ecological specialization; or early stages of speciation and divergence.

Previous work (Forsman, 2003; Baums et al., 2012; Boulay et al., 2014) has shown that corals long misidentified as varieties of *Porites lobata* from Panamá are actually *P. evermanni*, which is genetically, morphologically, and ecologically quite distinct from *P. lobata. P. evermanni* has been considered a Hawaiian endemic, however these studies have only recently shown that the geographic range of *P. evermanni* extends beyond Hawai‘i, and the true geographic range may be obscured by misidentification (Forsman, 2003; Boulay et al., 2014).

The goal of this study was to quantify genetic and morphological variation between species of *Porites* (*P. evermanni* vs *P. lobata*), relative to within species variation (*P. lobata*) across a broad geographic range. Genetic and morphological variation was characterized between colonies identified morphologically as *P. lobata* across a wide geographic range (the Galápagos, Easter Island, Tahiti, Rarotonga, Fiji, and Australia’s Great Barrier Reef). Using principal component discriminant analysis of skeletal micromorphological measurements, our goal was to test whether the landmarks could distinguish *P. evermanni* from *P. lobata*, and to examine within-species as opposed to between-species variation. In addition, we examined whether there was a relationship between the morphometric and genetic relationships between *Porites* across a broad geographic range.

## Materials and Methods

Small, fragments, ca. 10–15 g of tissue and skeleton were removed from colony edges, or protuberances, (in order to minimize damage to the donor colony) with the exception of Australia and Rarotonga where samples consisted of tissue scrapings with no skeletal voucher (Table 1). Samples were collected at least 10 m apart to minimize risk of collecting colonies that originated from clonal propagation or fragmentation. Samples were preserved in 95–100% ethanol. Specimens were compared to original type material from the Bernice Pauahi Bishop Museum under a dissecting microscope to confirm species identification. The samples were divided into several pieces when returned the laboratory; a small piece was stored in 95% ethanol at −20 °C for genetic analysis, and larger pieces were placed in household bleach to dissolve the soft tissue, prior to drying. Each skeletal fragment was approximately 2 to 5 cm^2^ containing between 5 and 40 corallites.

**Table 1:** Length variation, number of individuals, number of sequences, geographic region, collector and date for the ITS-1 and ITS-2 sequences collected for this study. Samples in bold letters indicate that a skeletal voucher specimen was collected.

					ITS-1	ITS-2
		Collector	No of	No of	Length	Length
Species	Region	(year)	individuals	sequences	(bp)	(bp)
*P. evermanni*	**Panama (Uva, Saboga)**	GMW(1999)	4	7	303–311	228–229
*P. lobata*	**Easter Island (La Perouse)**	GMW(1999)	4	10	303–312	215–226
” ”	Australia (One Tree Isl.)	MT (1998)	2	7	305–325	210–223
” ”	Rarotonga (Muri)	GMW(1999)	3	9	306–309	207–226
” ”	**Tahiti (Tikehau)**	GMW(1999)	3	9	306–309	207–231
” ”	**Galapagos (Wolf, Bartolome)**	ZHF (1998)	4	15	306–307	209–225
” ”	**Fiji (Namotu)**	GMW(1999)	3	7	305–309	215–223
		Total	23	64		

**Notes.**

MT M. Takabayashi GMW G. M. Wellington ZHF Z.H. Forsman

### Genetic analysis

DNA extraction, PCR, cloning and sequencing are described in detail elsewhere (Forsman, 2003); briefly, a few milligrams of tissue and skeleton were dried in a vacuum centrifuge for 20 min, the sample was then homogenized in a solution of 250 µl of 50 mM tris-HCL (pH 8.0) and 10 mM EDTA with a micro-pestle for 2 to 5 min. The homogenate was then frequently inverted during a 5 min room temperature incubation in 250 µl of 20 mM NaOH and 1% SDS. A volume of 350 µl of 3.0 M potassium acetate (pH 5.5) was added to the mixture and incubated for 5 min on ice followed by centrifugation at maximum speed. The top 500 µl of the cleared lysate was then transferred to a new tube and the DNA was precipitated by centrifugation in 1 ml isopropanol. The sample was then washed with 70% EtOH, dried and resuspended in 200 µl of H_2_O. The ITS region was amplified using the Eukaryotic ‘universal’ primers; ITS-1 (5’-TCC GTA GGT GAA CCT GCG G-3’) and ITS-4 (5’-TCC TCC GCT TAT TGA TAT GC-3’) (White et al., 1990) using the following PCR temperature profile: an initial denaturing period of 96 °C for 2 min followed by 30 cycles of: denaturing at 96 °C for 10 s, annealing at 50 °C for 30 s, and extension at 70 °C for 4 min, followed by a final 5 min extension step. PCR products were ligated into the PgemT-EZ cloning vector (Promega Inc.) and transformed into JM109 competent cells, followed by blue white colony screening. White colonies were screened for inserts, by colony PCR using the vector primers.

Each cloned sequence of the entire ITS region (ITS-1, 5.8S, ITS-2) was sequenced in both directions to ensure accuracy of each sequence. At least three individuals were sampled from each geographic region: Panamá, Galápagos, Easter Island, Tahiti, and Fiji, and at least 3 molecular clones were sequenced from each colony. Table 1 summarizes the geographic location of the samples collected, the collector, date of collection, and DNA sequence properties. The sequences have been deposited in GenBank under accession numbers: AY320289–AY320352. Sequence alignment was performed in ClustalW (Thompson, Higgins & Gibson, 1994), with a gap opening penalty [GOP] of 2, and a gap extension penalty [GEP] of 1. There were few alignment gaps or ambiguous positions and alternate alignments yield the same results (Forsman, 2003; Forsman et al., 2006). Previous work with the ITS region in *Porites* has shown that the marker is highly congruent with mitochondrial markers, although mitochondrial markers offer very little to no polymorphism at the species level (Neigel, Domingo & Stake, 2007; Shearer & Coffroth, 2008; Wares, 2014). While this sample size may be small for estimating population genetic structure, the purpose of this study was specifically to compare whether there is a relationship between genetic distance relative to morphological measurements of colonies across this broad geographic range.

**Table 2:** Definitions and descriptions of the morphological variables. See Fig. 1 for an illustration of the point landmarks.

Name	Points	Description	Dame	Description
SL1	1:2	Septa length	NP	Number of pali
SL2	3:4	Septa length	TRI	Triplet
SL3	5:6	Septa length	FA	Fossa area
SL4	7:8	Septa length	CA	Calyx area
SL5	9:10	Septa length	NR	Number of radi
SL6	11:12	Septa length		
SL7	13:14	Septa length	**Proportional variables**	
SL8	15:16	Septa length	FACA	FA/CA
SL9	17:18	Septa length	X1	(20:24 + 4:10)/(5:7 + 19:21)
SL10	19:20	Septa length	X2	24:4/23:3
SL11	21:22	Septa length	X3	SW/(1:2:13:14)
SL12	23:24	Septa length	X4	12:16/11:15
SW1	25:26	Septa width	X5	13:14/L
SW2	27:28	Septa width	X6	1:2/L
SD1	1:3	Septa distance	X7	23:3/L
SD2	3:5	Septa distance	LAT	3:5 + 7:9 + 17:19 + 21:23/L
SD3	5:7	Septa distance		
SD4	7:9	Septa distance		
SD5	9:11	Septa distance		
SD6	11:13	Septa distance	**Averaged variables**	
SD7	13:15	Septa distance	APD	Avg (PD)
SD8	15:17	Septa distance	ASL	Avg (SL)
SD9	17:19	Septa distance	ASW	Avg (SW)
SD10	19:21	Septa distance	IRR^*^	
SD11	21:23	Septa distance		
SD12	23:1	Septa distance		
PD1	2:4	Pali distance		
PD2	4:6	Pali distance		
PD3	6:8	Pali distance		
PD4	8:10	Pali distance		
PD5	10:12	Pali distance		
PD6	12:14	Pali distance		
PD7	14:16	Pali distance		
PD8	16:18	Pali distance		
PD9	18:20	Pali distance		
PD10	20:22	Pali distance		
PD11	22:24	Pali distance		
PD12	24:2	Pali distance		
FL1	20:8	Fossa width		
FL2	2:14	Fossa length		
W	7:19	Width		
L	1:13	Length		

**Notes.**

* IRR (septal irregularity) was calculated as the sum of the absolute value of the differences between septal lengths.

A distance tree was constructed for all 64 sequences using the Neighbor-Joining (Saitou & Nei, 1987) method (Fig. 2). Genetic distances (Table 3) were calculated using Kimura’s two-parameter model (Kimura, 1980). The tree was bootstrapped (1,000 replicates) and implemented in MEGA 2.1 (Kumar, Tamura & Nei, 1994). Data were robust to the tree-building algorithm, with Maximum Likelihood and Parsimony methods implemented in PHYLIP v3.6 (Felsenstein, 1989), and MEGA 2.1 yielding consistent relationships to the NJ tree. Sequences were grouped according to region, and the average distance within and between regions was calculated separately for each the ITS-1 and ITS-2 region in MEGA 2.0. Furthermore, a molecular analysis of variance (AMOVA) was implemented in Arlequin v 2.0 (Schneider, Roessli & Excoffier, 2000), with a transition/transversion weight of 2:1 and a gap weight of one (as with the relationships in the distance tree, alternative weighing schemes did not alter the outcome). Distances were calculated with the Kimura (1980) model, and a 0.2 gamma shape parameter (the shape parameter was estimated in PHYML v 1.0) (Guindon et al., 2009), by the maximum likelihood method implemented in the program. An AMOVA was performed on the entire data set (including all molecular clones except for one sequence that had several ambiguous positions), and then on separate subsets of each molecular clone per individual, in order to determine if the analysis was sensitive to differences in sample size, copy number, or haplotype identity within individuals and between populations. Each subset reflected highly significant genetic structure between geographic regions (Table 4). Significance tests were carried out with 10,023 permutations to generate a null distribution under the assumption of no genetic structure (Excoffier, Smouse & Quattro, 1992). Pairwise *F* statistics were calculated in Arlequin v 2.0 (Schneider, Roessli & Excoffier, 2000), and tested for significance by 10,023 permutations (Table 5).

**Figure 1:**
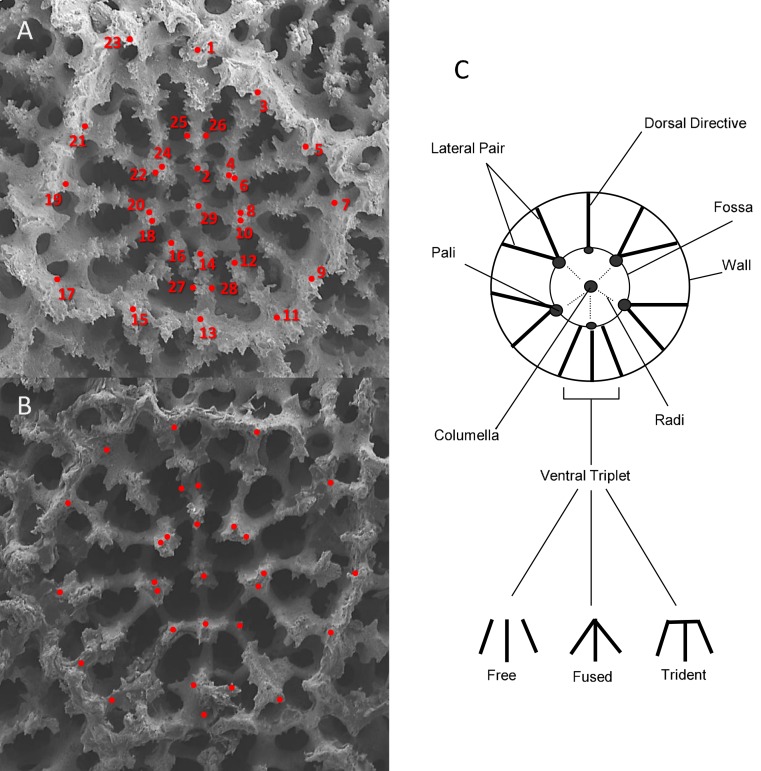
An illustration of the corallite morphometric characters used in this study. (A) SEM image of BPBM-SC454 *Porites lobata forma centralis* *β*, (Vaughan 1905 syntype; Oahu et al., 1904). (B) SEM image of BPBM-SC455 *Porites evermanni*, (Vaughan 1905 type; near Pearl Harbor Thompson 1904). (C) Schematic diagram of *Porites* primary diagnostic features.

**Figure 2:**
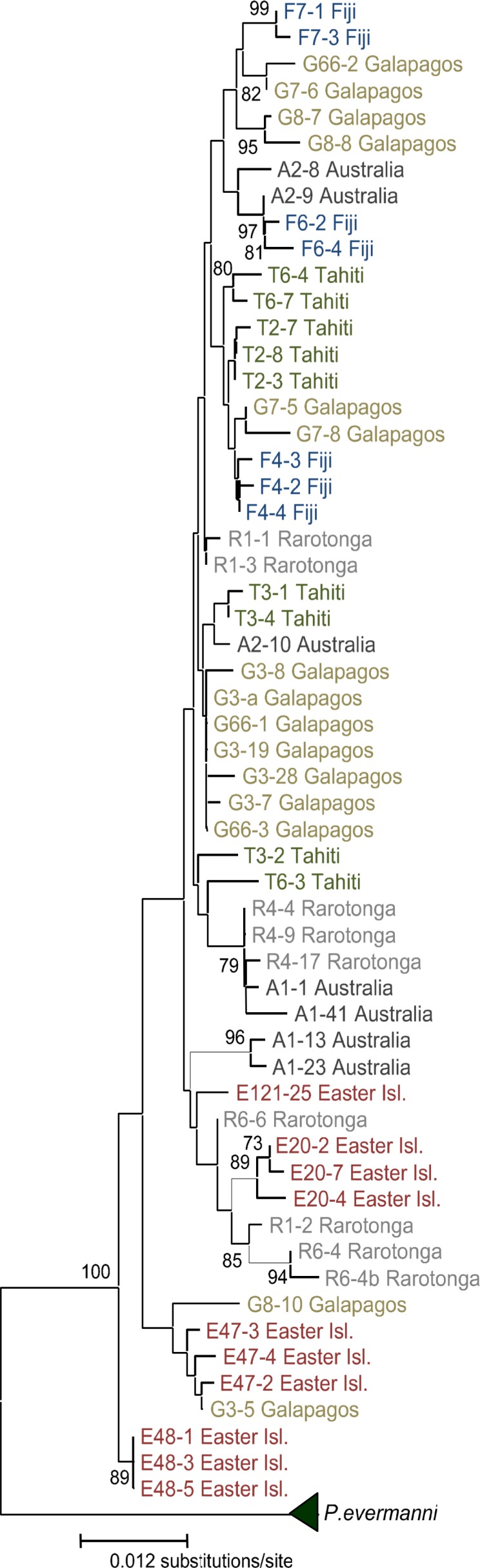
Neighbor-Joining tree of distances between all sequences in this study. Geographic regions sampled are indicated by color. The sample number is followed by a dash representing the number of the molecular clone. Bootstrap values below 70% are not shown. The triangle represents the collapsed *P.evermanni* clade; the height of the triangle is proportional to the genetic variability within the clade.

**Table 3:** Matrix of mean genetic distance between populations (in substitutions per site and standard errors, calculated by the Kimura, 1980 method). Standard errors are in italic script. Numbers along the diagonal represent intra-population means and standard errors.

	**Mean difference within and between groups**						
	Australia	Easter Isl.	Fiji	Galapagos	Rarotonga	Tahiti	*P. evermanni*
				**ITS-1**			
**Australia**	**0.012** ±*0.005*	*0.004*	*0.006*	*0.005*	*0.004*	*0.005*	*0.009*
**Easter Island**	0.013	**0.010** ±*0.004*	*0.006*	*0.005*	*0.004*	*0.005*	*0.010*
**Fiji**	0.018	0.019	**0.016** ±*0.006*	*0.005*	*0.005*	*0.005*	*0.010*
**Galapagos**	0.014	0.014	0.015	**0.011** ±*0.004*	*0.004*	*0.004*	*0.010*
**Rarotonga**	0.013	0.012	0.016	0.012	**0.009** ±*0.004*	*0.004*	*0.010*
**Tahiti**	0.014	0.013	0.016	0.012	0.012	**0.010** ±*0.004*	*0.010*
***P. eve***	0.027	0.033	0.034	0.030	0.033	0.030	**0.003** ±*0.002*
				**ITS-2**			
**Australia**	**0.019** ±*0.006*	*0.007*	*0.004*	*0.004*	*0.005*	*0.004*	*0.019*
**Easter Island**	0.029	**0.019** ±*0.005*	*0.006*	*0.006*	*0.006*	*0.006*	*0.019*
**Fiji**	0.016	0.024	**0.009** ±*0.003*	*0.004*	*0.004*	*0.003*	*0.018*
**Galapagos**	0.020	0.023	0.014	**0.016** ±*0.004*	*0.004*	*0.003*	*0.019*
**Rarotonga**	0.019	0.022	0.016	0.018	**0.015** ±*0.005*	*0.004*	*0.019*
**Tahiti**	0.014	0.024	0.010	0.013	0.015	**0.009** ±*0.003*	*0.019*
***P. evermanni***	0.110	0.114	0.106	0.108	0.117	0.108	**0.005** ±*0.002*

**Table 4:** AMOVA tables of genetic structure within and between geographic regions for *P. lobata*. (A) All sequences included. (B) One sequence per individual. (C) The most distinct population (Easter Island) excluded.

Source of variation	d.f.	S.S.	V.C.	% variation	
**(A) All sequences included**					
Among regions	5	59.115	(a) 0.89	19.79	*p* < 0.0001
Within regions	50	181.076	(b) 3.62	80.21	
Total	55	563.83	10.6		
**(B) One sequence per individual**					
Among regions	5	36.545	(a) 0.72	12.43	*p* < 0.02
Within regions	13	65.75	(b) 5.06	87.57	
Total	18	102.296	5.78		
**(C) The most distinct population (Easter Island) excluded**					
Among regions	4	29.38	(a) 0.44	11.78	*p* < 0.0001
Within regions	42	138.64	(b) 3.30	88.22	
Total	46	168.02	3.72		

**Table 5:** Pairwise *F_ST_* and significance values between geographic regions for *P. lobata*. Numbers in bold script represent statistical significance at or below the *α* = 0.05 level, cells are shaded darker for higher significance values.

	Easter Island	Australia	Rarotonga	Tahiti	Galapagos	Fiji
**Easter Isl**	∼	**0.0001**	**0.0001**	**0.0001**	**0.0001**	**0.0001**
**Australia**	**0.34**	∼	0.06	0.07	**0.01**	0.15
**Rarotonga**	**0.32**	0.12	∼	**0.01**	**0.001**	**0.01**
**Tahiti**	**0.36**	0.09	**0.15**	∼	**0.05**	**0.01**
**Galapagos**	**0.25**	**0.12**	**0.16**	**0.07**	∼	**0.05**
**Fiji**	**0.38**	0.07	**0.20**	**0.15**	**0.09**	∼

### Morphometric analysis

For each skeletal voucher, at least 3 digital images were captured at 18X magnification using a dissecting microscope attached to a digital CCD video camera, and a digital frame-capturing device (ATI all-in-wonder card; ATI technologies Inc.). A monofilament line of known thickness (0.16 mm) was used as a size reference for scaling each image. The images were scaled, and measured using the program Scion Image (Scion Corporation 2000). An average of ten corallites were measured for each individual voucher specimen (listed in Table 2) in order to obtain the average and range of morphometric measurements. The definitions of taxonomic characters are based on Veron & Stafford-Smith (2000) and Weil (1992), and are similar to two-dimensional characters previously used for species delimitation work in *Porites* (Budd, Johnson & Potts, 1994; Johnson & Budd, 1996; Jameson, 1997).

For each corallite, a series of 29 X-Y point coordinates were digitized according to prominent skeletal landmarks related to septal length and relative position, starting from the dorsal directive and proceeding in a clockwise fashion (depicted in Fig. 1). All landmarks were scored by a single observer (ZHF), with points placed either in the center of a feature (such as pali) or at the intersection between two features (such as septa and calice wall). The distance between each of the X-Y landmark coordinates was then calculated using the distance formula:

\begin{eqnarray*} \sqrt{({x}_{2}-{x}_{1})^{2}+({y}_{2}-{y}_{1})^{2}}. \end{eqnarray*}

Areas were estimated as polygons connecting the X-Y landmarks. For each corallite, 47 morphometric traits were measured; 42 linear measurements between selected point coordinates (Table 2, Fig. 1), 2 area measurements (fossa and calice area), and 3 discrete variables: (a) number of pali; (b) number of radi; and (c) ventral triplet margins fused, free, or tridented (Fig. 1). Nine additional morphometric variables were then calculated as proportions of combinations of linear measurements, and four were averages (Table 5). All raw measurements are presented in Table S1.

A forward stepwise discriminate analysis was implemented in Systat v.9 1998 (SPSS Inc.). All variables in Table 2 were initially included using automatic forward stepping with default options selected. The aim of the discriminate analysis was to find a linear combination of morphometric measurements that best discriminates between user-defined groups, i.e., species, or populations (Table 6 and Fig. 4). In order to examine the relationship between morphology and genetic distance among populations and species, distance matrices of averaged genetic distance and average morphological distance were compared using the Mantel test, as implemented in Arlequin v2.0. The significance tests of linear regressions of distance matrices are not reliable due to violations of assumptions of independence between data-points, therefore Mantel tests were used (Table 7 and Fig. 5). The Mantel test allows for autocorrelation within a matrix and tests for significant correlations between matrices by a permutation procedure (Mantel, 1967; Smouse, Long & Sokal, 1986).

**Figure 3:**
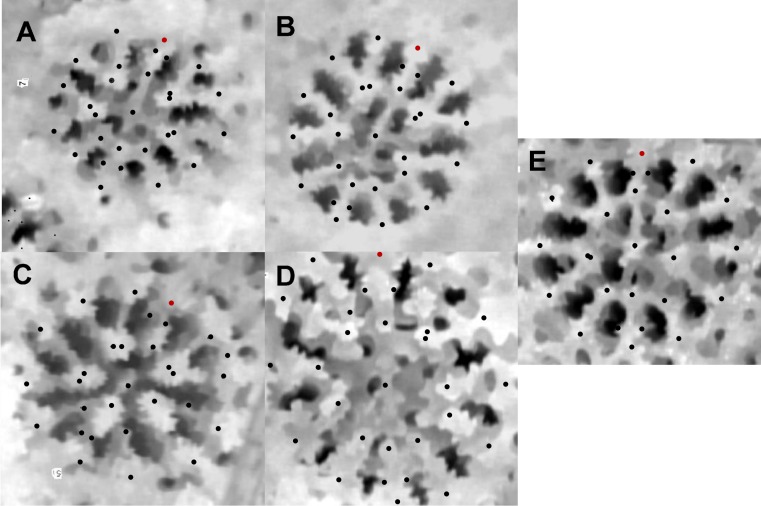
An example of geographical variation among *P. lobata spp*. (A) Tahiti, (B) Galapagos, (C) Fiji, (D) Easter Island, (E) *P. evermanni*. The dorsal directive is indicated by a red dot in the upper area of the image.

**Figure 4:**
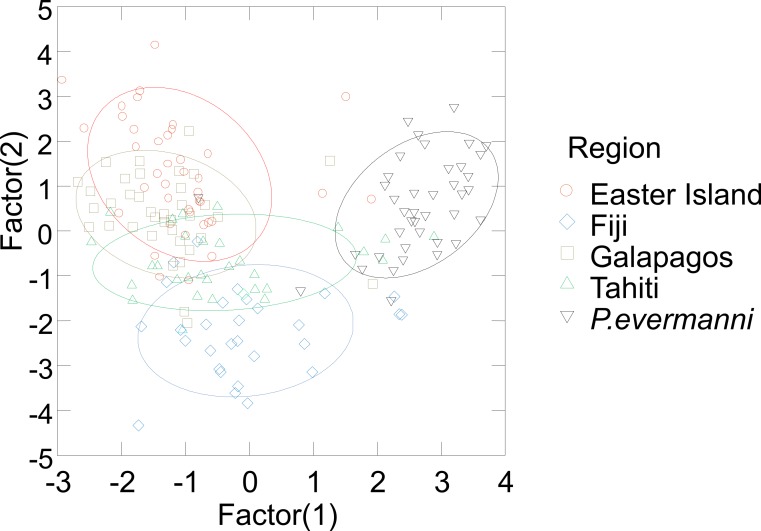
Stepwise multivariate cononical discriminant analysis plot of the two factors with the largest covariance. 95% confidence ellipses are drawn around the data from each region.

**Figure 5:**
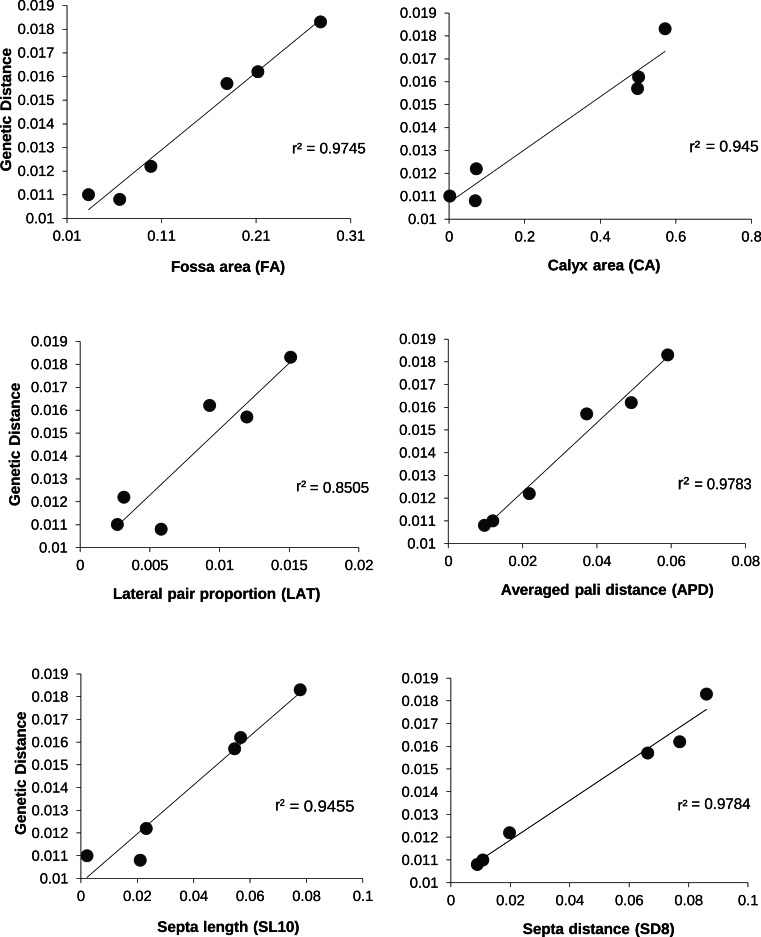
The relationship between genetic and morphologic distances between *P. lobata* populations. The *r*^2^ value for a linear regression are indicated.

**Table 6:** Jackknifed classification matrix. The Jackknifed classification matrix indicates how many corallites were correctly classified by groupings based on regions (e.g., 95% of *P. evermanni* corallites were classified correctly). The eigen values indicate that the first two factors account for the largest portion of the variance.

	Eeaster Island	Fiji	Galapagos	Tahiti	*P. evermanni*	% correct
**Easter Island**	26	2	5	4	3	65
**Fiji**	0	19	1	7	3	63
**Galapagos**	7	1	23	4	2	62
**Tahiti**	3	3	3	16	5	53
***P. evermanni***	0	0	1	1	38	95
**Total**	**36**	**25**	**33**	**32**	**51**	**69**
		**Eigen values**				
		2.255	1.427	0.351	0.229	

**Table 7:** Morphological variables that had a significant relationship to genetic distance (see Fig. 5 for examples). Values highlighted in bold font were significant at < 0.05 level according to Mantel tests. The abbreviations of morphologic characters are listed in Table 2.

Variable	*r* ^2^	Significance
SL5	0.80	*
SL6	0.89	**
SL7	0.83	**
**SL8**	**0.95**	***
SL10	0.68	*
SL11	0.66	*
SD1	0.88	**
SD3	0.90	**
SD4	0.75	*
SD5	0.90	**
**SD6**	**0.92**	**
SD7	0.80	*
**SD8**	**0.98**	***
SD9	0.85	**
**SD10**	**0.95**	***
SD11	0.72	*
SD12	0.85	**
PD2	0.85	**
PD3	0.79	*
PD4	0.82	*
**PD5**	**0.97**	***
PD6	0.87	**
PD7	0.81	*
**PD8**	**0.98**	***
FL1	0.90	**
FL2	0.75	*
**PD10**	**0.94**	***
PD12	0.78	*
**W**	**0.96**	***
**L**	**0.92**	**
**FA**	**0.97**	***
**CA**	**0.95**	***
X1	0.66	*
LAT	0.85	**
**APD**	**0.98**	***

**Notes.**

* *p* < 0.05 ** *p* < 0.01 *** *p* < 0.001

## Results

### Genetic analysis

The Panamanian *P. evermanni* samples were genetically distinct from all *P. lobata* specimens collected across the broad geographic range (Galápagos, Easter Island, Tahiti, Fiji, Rarotonga, and Australia). The majority of molecular clones from the same specimen were very similar, and clustering typically occurred between sequences from the same individual (Fig. 2). Within-population ITS variability was lower than between-population variation, particularly in the ITS-2 region (Table 3). However, differences between species were an order of magnitude larger than within species variation. *P. evermanni* had at least two or three times lower within-species variation than any populations of *P. lobata* sampled (Table 3).

In order to determine if significant geographic structure occurred between the populations sampled, a molecular analysis of variance (AMOVA) was performed (Excoffier, Smouse & Quattro, 1992). Differences between geographic regions were significant (*p* < 0.0001), with nearly 20% of the molecular variance attributed to differences between regions (Table 4). This result was robust to both using a single sequence per individual (a similar result was obtained with each of the three different pairwise combinations of molecular clones), and also to the exclusion of the most distinct population (Easter Island) from the analysis (Table 4). Thus, significant geographic structure is not due solely to the inclusion of a single genetically distant group (Easter Island), nor to sampling unequal numbers of sequences per individual or per region. Despite low sample sizes, pairwise *F_ST_* values indicated that there was significant genetic structure between most of the populations, with the exceptions of a few geographic regions, particularly comparisons with Australia (Table 5). The most geographically isolated island (Easter Island) was the most genetically distinct from all regions, followed by the Galapagos Archipelago and Rarotonga.

### Morphometric analysis

Corallites generally appeared to vary by region (Fig. 3), and the majority of measured traits exhibited significant differences among geographic regions. Based on ANOVA and on Tukey’s HSD post-hoc comparisons, nearly all measurements showed significant differences between some regions (Panamá followed by Easter Island were most frequently distinct). The stepwise canonical discriminant analysis indicated that *P. evermanni* from Panamá were consistently distinguishable from *P. lobata* (Wilks’ lambda = 0.076, *p* < 0.0001, Fig. 4). The variables TRI (triplet free, fused, or trident), NP (number of pali), W/L (calice width divided by calice length), X6 (distance between dorsal lateral pairs divided by calice length), FA (fossa area), and CA (calice area) had the largest influence on discriminating between species. The jackknifed classification matrix indicates how many corallites were correctly classified by groupings based on regions, showing that 95% of Panamá region corallites were classified correctly (Table 6). The eigenvalues indicate that the first two factors account for the largest portion of the variance. As might be expected, neighboring populations tended to overlap in the morphometric analyses more than populations at extreme ends of the geographic range (for example, Galápagos and Fiji were nearly completely non-overlapping).

Linear regressions of the average genetic distances between populations of *P. lobata* with the average morphologic distances between populations were significant for 35 of the 42 variables measured (83%; Table 7); however, due to non-independence among variables, and likely co-linearity among these landmark measurements, we employed the more conservative Mantel test, which indicated that 12 of the 42 morphometric variables (29%) vary significantly with the average genetic distance between regions (Table 7 and Fig. 5).

## Discussion

As coral reef ecosystems face global decline, there is an increased need to evaluate extinction risk, and to map changes in species distributions for both science and policy (Brainard et al., 2011). For species which are difficult to distinguish, such as *Porites*, this task is particularly daunting. Species misidentification can confound a variety of studies that involve this ubiquitous architect of coral reefs. *Porites* is a model organism for paleoclimate work because colonies deposit annual growth bands that preserve the chemical signatures of the sea and they are among the world’s oldest animals (Brown et al., 2009). The *Porites* fossil record is extremely well preserved, particularly the microscopic features that were the central focus of this study. Clarification of molecular and morphological variation between and within species therefore has important implications for understanding the past, present, and future of coral reefs and the tropical seas they inhabit. This study found that both molecular and morphometric tools readily distinguish two massive corals (*P. lobata* and *P. evermanni*) that are difficult to distinguish *in-situ*. Both methods also revealed significant geographic variability in *P. lobata* sampled across the Eastern and Central Pacific Ocean, consistent with previous studies (Baums et al., 2012; Polato et al., 2010).

The two species (*P. lobata* and *P. evermanni*) were reciprocally monophyletic with strong statistical support (Fig. 2), which was found by previous studies (Forsman et al., 2009; Boulay et al., 2014). Each population of *P. lobata* had nearly double the genetic variability of *P. evermanni* sampled from Panama (Table 3). This pattern of reduced genetic variation may be due to isolated population of *P. evermanni* in Panama, assuming that the complex evolutionary history of the multicopy ITS region is comparable in both species (Forsman et al., 2006; Coleman, 2009; Stat et al., 2012). Previous work using microsatellite loci also found lower levels of genetic variation in *P. evermanni* than *P. lobata*, as well as major ecological differences between species (Boulay et al., 2014). The two species were also found to have key ecological differences such as higher susceptibility of *P. lobata* to coral bleaching and further distribution from the shore, while *P. evermanni* was observed to reproduce asexually via fragmentation from triggerfish bites (Boulay et al., 2014).

Within *P. lobata*, the ITS region showed significant genetic structure, with approximately 20% of the variation due to differences between populations. The most geographically isolated population (Easter Island) was the most genetically distinct population (Table 3). Although Easter Island was the most genetically distinct population, the genetic differences were on a scale consistent with intraspecies variation (1–2% for ITS-2), whereas differences from the congeneric *P. evermanni* was an order of magnitude higher (10–11% for ITS-2). *Porites* colonies from Easter Island have long been recognized as having a distinct appearance and they were initially described as a separate species, *P. paschalensis* (Vaughan 1906), which is now considered a junior synonym of *P. lobata* (Wells, 1972; Glynn et al., 2007). Easter Island *P. lobata* colonies tend to form tall columnar fins or peaks, and tend to have large open irregular corralites (Glynn et al., 2007). Pairwise *F_ST_* comparisons with Easter Island were higher than any other location, and all highly significant (Table 5). The Galapagos archipelago also had significant genetic structure when compared to all other populations, which is consistent with previous work that has found barriers between the Eastern Tropical Pacific, and the Central Tropical Pacific biogeographic zones (Baums et al., 2012). Interestingly, all other populations (with the exception of most comparisons with Australia) also show significant genetic structure. The overall pattern is consistent with expectations from geographic isolation-by-distance. Previous work has found geographic isolation-by-distance in *P. lobata* across the Hawaiian archipelago, and reduced gene flow between Hawaii and Johnston atoll, separated 2,500 km away (Polato et al., 2010). Patterns of isolation-by-distance were also found in *P. lobata* sampled across larger oceanographic scales, with major genetic breaks found between isolated biogeographic zones, particularly Hawai‘i and the Eastern Pacific (Baums et al., 2012). When combined, these studies indicate that there are major oceanic barriers to gene flow, and that remote populations such as Easter Island are particularly genetically isolated and therefore more vulnerable and reliant on self-recruitment for recovery after disturbance.

Multivariate coralite level measurements were also effective for distinguishing inter- and intraspecific variation. Multivariate measurements alone distinguished between *P. lobata* and *P. evermanni* for 95% of the coralites compared (Table 6). According to these measurements, each *P. lobata* population most closely resembled its nearest geographic neighbor, and in the majority of cases (53–65%) individual corralites could be identified to the correct population (Table 6). This result suggests that geographic differences are likely to overshadow effects of within colony variability and morphological variation across habitats; however, these effects were not examined in this study and the topic should be fertile ground for further work. The ability to distinguish morphological variability will most likely be increased by the addition of additional characters such as corralite wall and pali height (or 3D measurements) because these characters are also considered diagnostic for some *Porites* species (Veron & Stafford-Smith, 2000). Previous work successfully used 3D landmarks to distinguish Caribbean *Porites* (Budd, Johnson & Potts, 1994; Johnson & Budd, 1996). The simple and rapid two-dimensional coralite-level measurements used in this study are very similar to previously used landmarks, and they appear to correlate well with genetic distance (Table 7 and Fig. 5), particularly many of the measurements of the length of the ventral triplet, or the distance between ventral triplet pali appear to be particularly informative (Table 7 and Fig. 5), features that have long been suspected of being diagnostic and informative characters for the genus (Bernard, 1902; Jameson, 1997; Veron & Stafford-Smith, 2000).

This study illustrated that easily misidentified *Porites* samples can be distinguished through genetic sequence data, morphological measurements, or a combination of both. Both molecular and morphometric methods were further able to reveal congruent population-level differentiation. The concordance between genes and mircro-morpholgy found in this and other studies (Budd, Johnson & Potts, 1994; Budd et al., 2010; Luck, Forsman & Toonen, 2013; Marti-Puig et al., 2014) lends weight to both as reasonable characters for the determination of lineages and gives hope for new taxonomic characters to help resolve the species problem in corals. The ITS region clearly differentiates these difficult to identify species; however, direct sequencing results in poor chromatogram quality and molecular subcloning was needed for this study, which is expensive and time consuming. Unless additional informative molecular markers are developed for *Porites*, ITS sequence data can be used to develop more rapid and low cost assays such as restriction fragment length polymorphism (RFLP) or species specific PCR probes or primers. Likewise, now that informative morphometric characters have been identified, further work can develop rapid and more automated methods of detecting and measuring skeletal landmarks in much the same way that fingerprint or other biometric data is scanned to rapidly identify individual human beings. Such biometric technology would prevent misidentification that can greatly confound a wide variety of studies, while providing new biological insights that would otherwise be obscured. Confident species identification would at the very least: (1) improve species geographic and habitat distribution maps, (2) allow changes in species distributions to be monitored (3) improve evaluation of rare or endangered species (4) allow extinction to be monitored (5) improve the understanding of ecological interactions (6) improve the understanding of resilience and sensitivity to disturbance for a given species (7) improve paleoclimate studies, and (8) assist with interpreting the fossil record. A combined approach that integrates both molecular and morphometric data is an important first step towards understanding the complex history of coral species in space and time.

## Supplemental Information

10.7717/peerj.751/supp-1 Table S1 Raw morphometric measurements
